# Supplementary material for: Membranes prime the RapGEF EPAC1 to transduce cAMP signaling
Source: Nat Commun. 2023 Jul 12;14:4157. doi: 10.1038/s41467-023-39894-4 (PMC10338474; doi:10.1038/s41467-023-39894-4)
Supplement: Supplementary file 1 — Supplementary Information [file 41467_2023_39894_MOESM1_ESM.pdf]

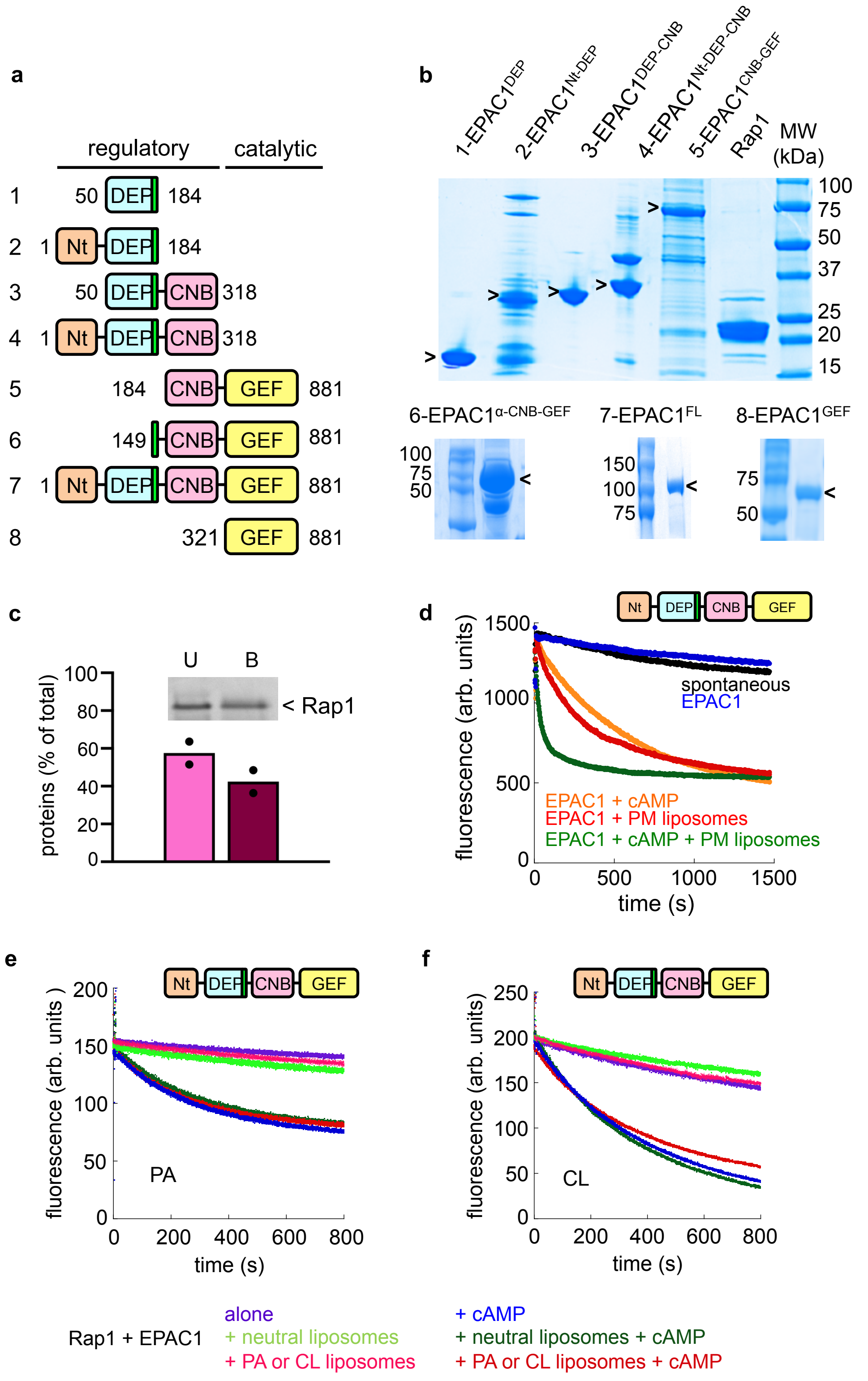

Supplementary Fig. 1

Supplementary Fig. 1 Membranes directly activate EPAC1^{FL} and increase its affinity for cAMP. a EPAC1 constructs used in this study. **b** Representative SDS-PAGE and Coomassie blue staining of the purified proteins used in this study, indicated by “>”. Molecular weight markers (MW, in kDa) are in the right lane (upper gel) or the left lane (bottom gel). **c** Non-lipidated full-length Rap1 binds to PM liposomes. Binding was analyzed by liposome flotation. Top, representative Coomassie blue staining of bound (B) and unbound (U) protein. Bottom, diagram showing % bound and unbound protein as mean of n = 2 independent experiments. **d** Representative fluorescence kinetics showing the activation of Rap1 by EPAC1^{FL} in solution or on liposomes, with or without cAMP. Related to **Fig. 1c**. **e, f** Representative fluorescence kinetics showing the activation of Rap1 by EPAC1^{FL} on neutral liposomes supplemented with 10% PA (**e**) or 10% CL (**f**). The colour scheme is the same for both panels, as indicated below the diagrams. Related to **Fig. 1d** and **1e**.

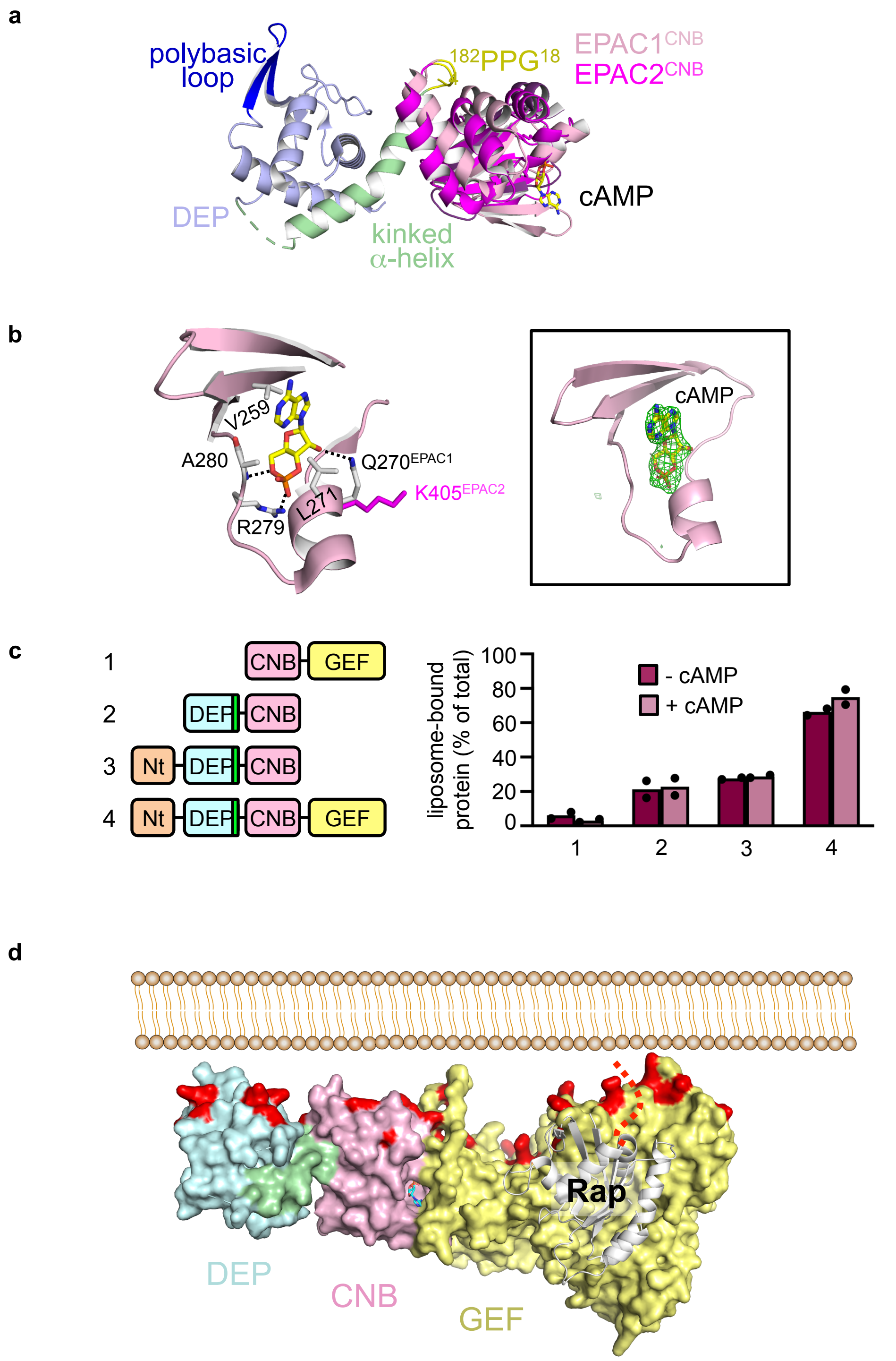

Supplementary Fig. 2

Supplementary Fig. 2 Structural determinants of EPAC1 binding to membranes.

a Comparison of EPAC1$^{DEP-CNB}$ and EPAC2$^{DEP-CNB}$. The superimposition is based on the DEP domain/kinked α-helix, which is shown only once for clarity. The DEP domain/kinked α-helix moieties and the CNB domains are similar, but display a small difference in their respective orientations. The CNB domain of EPAC1 is in light pink, the CNB domain of EPAC2 in bright pink. The hinge between the kinked helix and the CNB domain is in yellow. EPAC2 is from the structure of the regulatory domain (PDB entry 1O7F). **b** The cAMP-binding site of EPAC1$^{DEP-CNB}$. EPAC1 and EPAC2 differ at Q270 (EPAC1) and K405 (EPAC2). Hydrogen bonds are shown as dotted lines. The omit map showing the electron density of cAMP contoured at 5σ is shown in inset. **c** cAMP does not increase binding of CNB domain-containing EPAC1 constructs to PM liposomes. Binding was measured by liposome flotation. Left, EPAC1 constructs used. Right, percentage of bound protein represented as mean of n = 2 independent experiments. **d** Docking of the composite EPAC-cAMP-Rap1 model (N-terminal domain not modelled) to membrane, predicted by the OPM server. Lysines and arginines are shown in red. The predicted position of the lipidated polybasic C-terminus of the Rap1 GTPase is shown as a red dotted line.

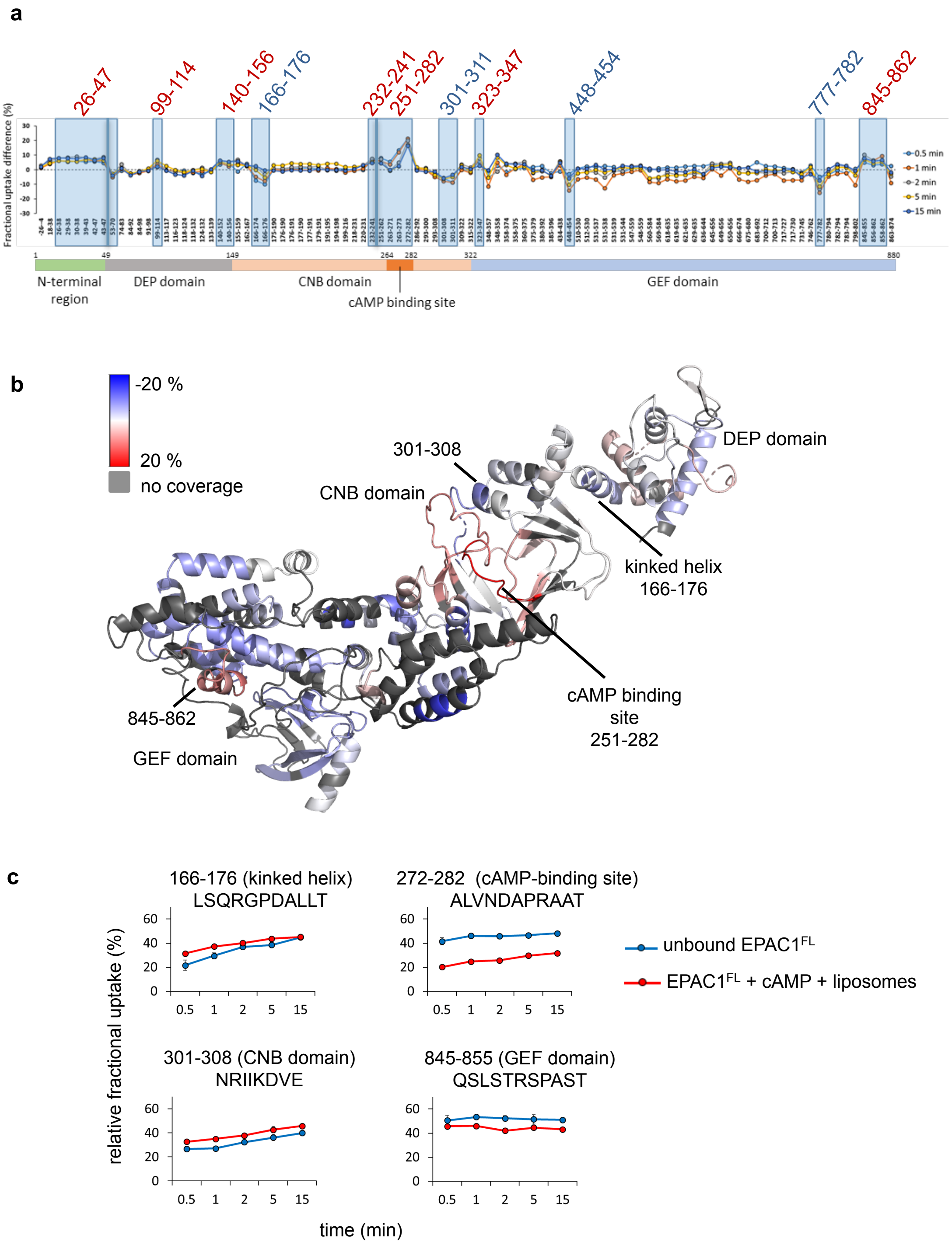

Supplementary Fig. 3

Supplementary Fig. 3 HDX-MS analysis of the activation of EPAC1 by cAMP and PM liposomes. a HDX-MS difference plots between unbound EPAC1 and EPAC1 bound to cAMP and liposomes. The difference plots illustrate differential deuterium uptake for each identified peptide at different incubation times: 0.5 min (cyan), 1 min (orange), 2 min (grey), 5 min (yellow) and 15 min (blue). Statistically significant differences in deuterium uptake (see Methods) are highlighted by blue boxes. **b** Relative deuterium uptake differences mapped onto the model of cAMP- and liposome-bound (fully active) EPAC1. Red: regions more protected in fully active EPAC1; blue: regions deprotected in fully active EPAC1; grey: regions with no peptide coverage. Fully active EPAC1 proved to be highly dynamic, with the N-terminal, CNB and GEF domains being either more (GEF) or less (N-terminal region, CNB) solvent-accessible. Notably, several regions of the GEF domain exhibited increased D incorporation at 1, 2 and 5 min while no differences were observed at 0.5 and 15 min. This is consistent with concurrent remodeling of intramolecular interactions and establishment of protein/membrane interactions as predicted in the model of fully active EPAC1. **c** Deuterium uptake graphs of representative peptides showing significant differences in deuterium uptake. Blue : unbound EPAC1; red: EPAC1-cAMP-liposomes. Related to Fig. 3d.

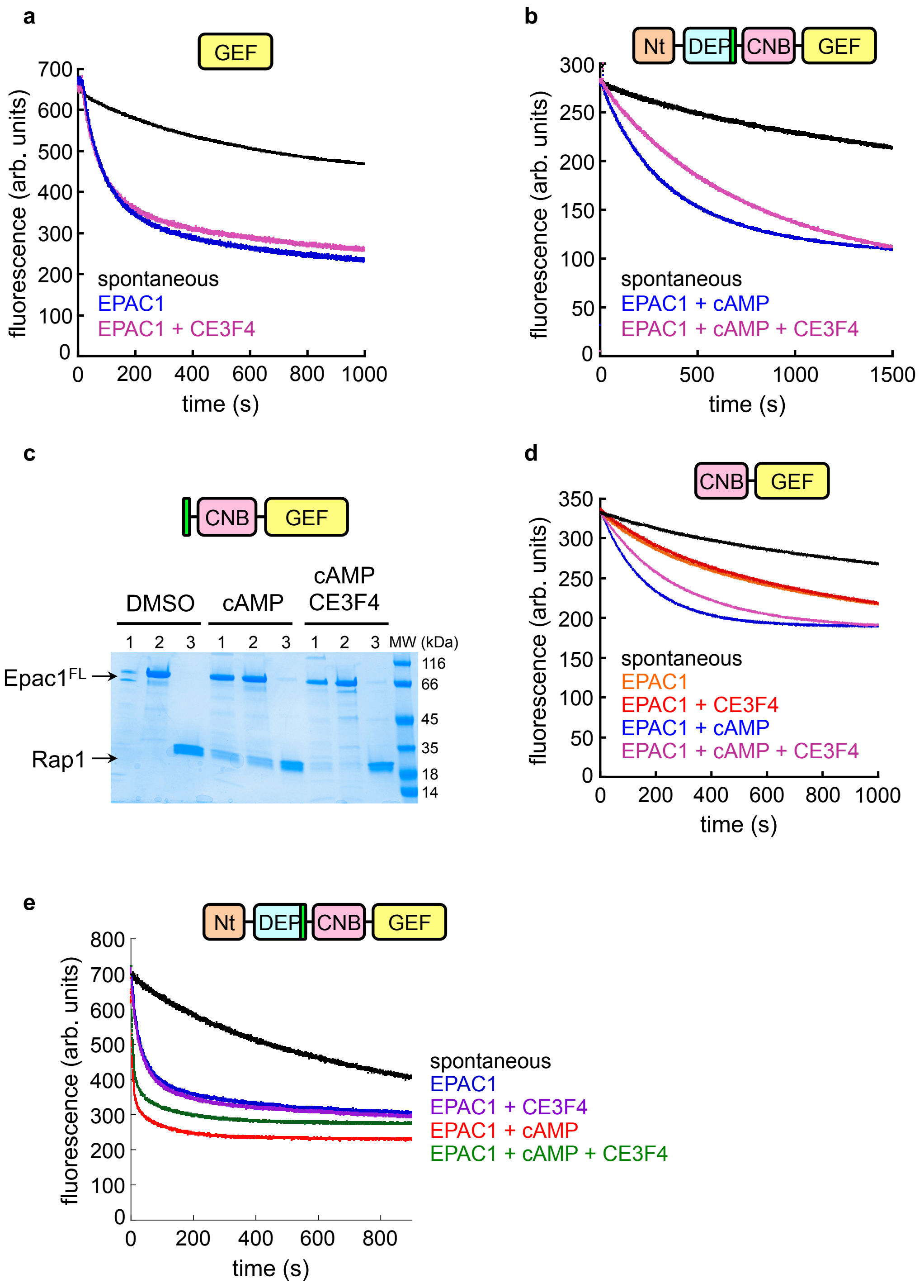

Supplementary Fig. 4

Supplementary Fig. 4 Biochemical inhibitory mechanisms of CE3F4. a CE3F4 does not inhibit $EPAC1^{GEF}$. Representative fluorescence kinetics in solution are shown. Kinetics were carried out in the same conditions as in Fig. 4a, except that $EPAC1^{FL}$ was replaced by $EPAC1^{GEF}$. **b** Representative fluorescence kinetics showing the inhibition of $EPAC1^{FL}$ by CE3F4 in solution. Related to Fig. 4a. **c** CE3F4 impairs the formation of the Rap1/$EPAC1^{\alpha\text{-CNB-GEF}}$ complex. The peak fractions of the size exclusion chromatography experiment shown in Fig. 4c were analyzed by SDS-PAGE. 1: EPAC1/Rap1 complex; 2: unbound EPAC1; 3: unbound Rap1. **d** Representative fluorescence kinetics showing the effect of cAMP on the inhibition of constituvely active $EPAC1^{CNB\text{-}GEF}$ construct by CE3F4 in solution. Kinetics were carried out as in Fig. 4a, except that $EPAC1^{FL}$ was replaced by $EPAC1^{CNB\text{-}GEF}$. Related to Fig. 4d. **e** Representative fluorescence kinetics showing the effect of cAMP on the inhibition of $EPAC1^{FL}$ by CE3F4 in the presence of PM liposomes. Kinetics were carried out as in Fig. 4a, except that PM liposomes were added. Related to Fig. 4e. All control experiments without CE3F4 were performed in the presence of vehicle (DMSO).

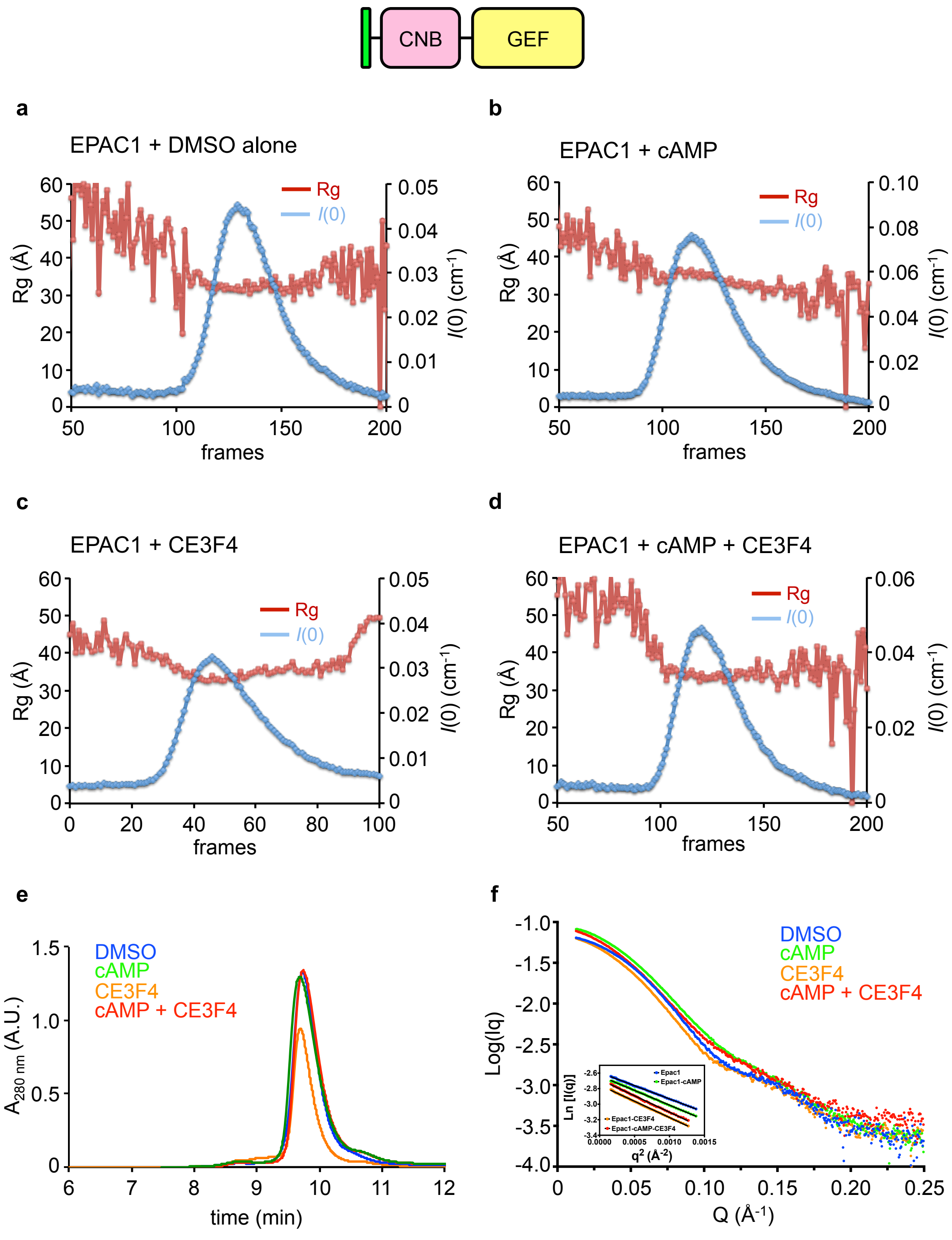

Supplementary Fig. 5

Supplementary Fig. 5 SEC-SAXS analysis of the inhibition of EPAC1$^{\alpha\text{-CNB-GEF}}$ by CE3F4.

a-d SEC-SAXS elution profiles of EPAC1$^{\alpha\text{-CNB-GEF}}$ in unbound state (**a**), cAMP-bound state (**b**), CE3F4-bound state (**c**) and cAMP- and CE3F4-bound state (**d**). EPAC1$^{\alpha\text{-CNB-GEF}}$ elutes as a single, well-resolved *I*(0) peak with stable radius of gyration (Rg, an indicator of the overall spread of the protein) in all conditions. Intensities are in blue (scale on the right), Rg are in red (scale on the left). **e** SEC-HPLC elution profiles. **f** SAXS scattering curves. All control experiments without CE3F4 were performed in the presence of vehicle (DMSO). Related to **Fig. 4b**.

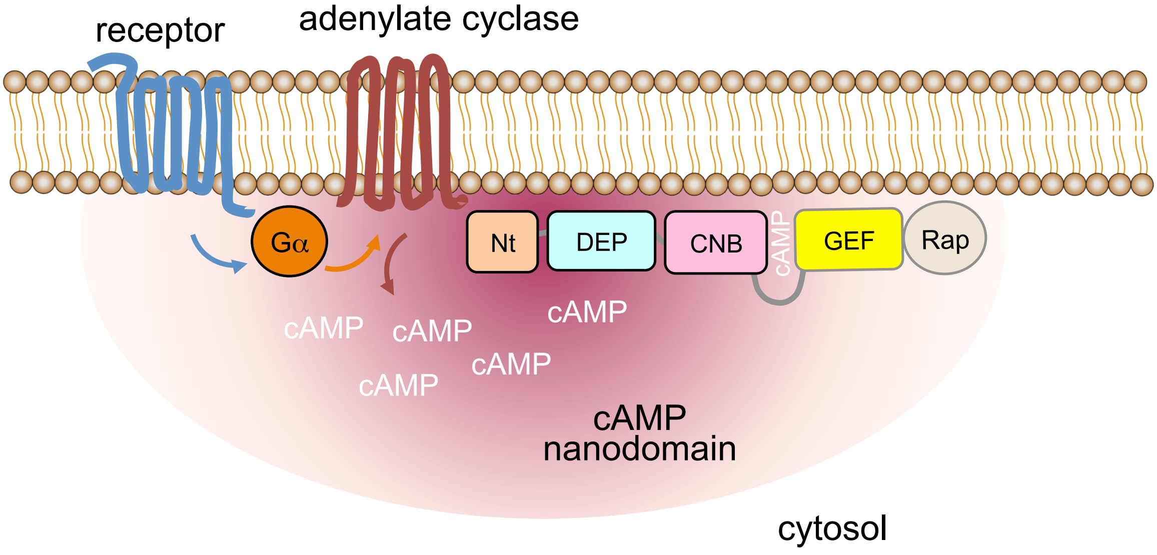

Supplementary Fig. 6 Membrane-attached EPAC1 has determinants for cAMP nanodomain signaling. The cAMP nanodomain proximal to the activated receptor and adenylate cyclase is indicated as a pink circle.

Supplementary Table 1.
X-ray crystallography structure of EPAC1$^{DEP\text{-}CNB}$-cAMP. Data collection and refinement statistics (molecular replacement)

	EPAC$^{DEP\text{-}CNB}$-cAMP
**Data collection**	
Space group	I121
Cell dimensions	
a, *b*, *c* (Å)	41.70 105.6 160 .56
α, β, γ (°)	90.00 90.04 90.00
Resolution (Å)	88.24–2.30 (2.39–2.30)*
R_{merge}	0.106 (0.9)
$I / \sigma I$	9.4 (1.95)
Completeness (%)	99.4 (98.9)
Redundancy	6.7 (6.8)
**Refinement**	
Resolution (Å)	2.3
No. reflections	30693
R_{work} / R_{free}	0.202/0.240
No. atoms	4111
Protein	3922
Ligand/ion	76
Water	135
B-factors	53.14
Protein	53.52
Ligand/ion	46.7
Water	44.69
R.m.s. deviations	
Bond lengths (Å)	0.007
Bond angles (°)	1.18

*Values in parentheses are for highest-resolution shell.

Supplementary Table 2.
SAXS analysis of EPAC1$^{\alpha\text{-CNB-GEF}}$. Data collection and analysis. Scattering curves were obtained by averaging frames corresponding to the high-intensity fractions of the peak and having constant radius of gyration (R_g) within error. Apo: frames 119-132; cAMP: frames 116-124; CE3F4: frames 112-121; cAMP-CE3F4 : frames 41-50.

**(*a*) Sample details**				
	Apo	+cAMP	+CE3F4	+cAMP +CE3F4
Organism	*Homo sapiens*			
Uniprot ID	O95398			
Extinction coefficient ε	75400 M^{-1} cm^{-1}			
Partial specific volume $\bar{\upsilon}$	102203.9$Å^3$			
Molecular mass *M*	82.2 kDa			
Loading volume/concentration	5 mg/mL			
Injection volume, flow rate	50 µl 0.2 ml/min			
**(*b*) SAXS data collection parameters**				
SWING beamline, equipped with a PCCD170170 Aviex Detector				
Wavelength 1.033 Å				
Beam geometry size, sample-to-detector distance 41.7 mm, 1994mm Pinhole				
q-measurement range $0.01 < q < 0.5$ $Å^{-1}$				
Exposure time, number of exposures 0.750s 180frame				
Sample temperature 20 ºC				
**(*c*) Software employed for SAXS data reduction, analysis and interpretation**				
SAXS data reduction to sample–solvent scattering, and extrapolation, merging, desmearing *etc*. as relevant : Foxtrot 3.2.7				
Calculation of ε from sequence : Expasy ProtParam tool				
Calculation of $\Delta\bar{\rho}$ and $\bar{\upsilon}$ values from chemical composition : NucProt Calculator				
Basic analyses: Guinier, *P*(*r*), scattering particle volume (*e.g.* Porod volume V_P or volume of correlation V_c) : ATSAS programs				
**(*d*) Structural parameters**				
Guinier Analysis	Apo	+cAMP	+CE3F4	+cAMP +CE3F4
I(0) (cm^{-1})	0.041	0.074	0.031	0.045
R_g (Å)	31.93	34.91	32.58	33.41
P(*r*) analysis				
I(0) (cm^{-1})	0.040	0.073	0.031	0.045
R_g (Å)	31.65	35.53	32.86	33.84
d_{max} (Å)	103	130	114	118
Molar mass (kDa) from Qp	86.9	95.9	91.2	85.3
Molecular mass (kDa) from MoW	90.3	99.5	92.3	91.0
Molecular mass (kDa) from Vc	82.7	88.3	86.0	84.4
Volume (*e.g.* V_P and/or V_c)	131000	140000	140000	134000

Supplementary Table 3.

Primers sequences.

Gateway cloning			
Destination Vector	Entry Vector	Primer	Sequence
pHMGWA Nt-DEP	pDONR221 Nt-DEP	AttB1_TEV_Nter_1	GGGGACAAGTTTGTACAAAAAAGCAGGCTTAGAAAACCTGTATTTTCAGGGCATGGTGTTGAGAAGGATGCACCGG
		AttB2_stop_DEP_184	GGGGACCACTTTGTACAAGAAAGCTGGGTTTATTAACCTGGGGGCTTTCGAAGTGCCAC
pHMGWA DEP	pDONR221 DEP	AttB1_TEV_DEP_50	GGGGACAAGTTTGTACAAAAAAGCAGGCTTAGAAAACCTGTATTTTCAGGGCGCCTCCACAGAGCGGGTGCTCAGG
		AttB2_stop_DEP_184	GGGGACCACTTTGTACAAGAAAGCTGGGTTTATTAACCTGGGGGCTTTCGAAGTGCCAC
pHMGWA Nt-DEP-CNB	pDONR221 Nt-DEP-CNB	AttB1_TEV_Nter_1	GGGGACAAGTTTGTACAAAAAAGCAGGCTTAGAAAACCTGTATTTTCAGGGCATGGTGTTGAGAAGGATGCACCGG
		AttB2_stop_CNB_318	GGGGACCACTTTGTACAAGAAAGCTGGGTTTATTAGCCATGTTCTTCCAGCCGCATGGT
pHMGWA CNB-DEP	pDONR221 CNB-DEP	AttB1_TEV_DEP_50	GGGGACAAGTTTGTACAAAAAAGCAGGCTTAGAAAACCTGTATTTTCAGGGCGCCTCCACAGAGCGGGTGCTCAGG
		AttB2_stop_CNB_318	GGGGACCACTTTGTACAAGAAAGCTGGGTTTATTAGCCATGTTCTTCCAGCCGCATGGT
pHMGWA alpha-CNB-GEF	pDONR221 alpha-CNB-GEF	AttB1_TEV_alphaCNB _149	GGGGACAAGTTTGTACAAAAAAGCAGGCTTAGAAAACCTGTATTTTCAGGGCCCCGTGGGAACTCATGAGATGG
		AttB2_stop_GEF_881	GGGGACCACTTTGTACAAGAAAGCTGGGTTTATTATCATGGCTCCAGCTCTCGGGAGAGG
pHMGWA CNB-GEF	pDONR221 CNB-GEF	AttB1_TEV_CNB_184	GGGGACAAGTTTGTACAAAAAAGCAGGCTTAGAAAACCTGTATTTTCAGGGCCAGCGCACGGATGAAGAGCTGGACC
		AttB2_stop_GEF_881	GGGGACCACTTTGTACAAGAAAGCTGGGTTTATTATCATGGCTCCAGCTCTCGGGAGAGG
pETG20 -GEF	pDONR221 -GEF	AttB1_TEV_GEF _321	GGGGACAAGTTTGTACAAAAAAGCAGGCTTAGAAAACCTGTATTTTCAGGGC GTGCTGGTGCTGGAGAGAGCCTC
		AttB2_stop_GEF_881	GGGGACCACTTTGTACAAGAAAGCTGGGTTTATTATCATGGCTCCAGCTCTCGGGAGAGG

Cloning by restriction enzymes		
Pet28a Epac1fL	fwd_nde1_TEV_epac1fl	GGGAATTCCATATGGAAAACCTGTATTTTCAGGGCATGGTGTTGAGAAGGATGCACCGGC
	Rev_not1_stop_epac1fl	TTTTCCTTTTGCGGCCGCTTATCATGGCTCCAGCTCTCGGGAGAGGCG
Pet3a Rap1a	fwd_ndeI_ATG_Rap1	GGGAATTCCATATGCGTGAGTACAAGCTAGTGGTCC
	Rev_BamHI_Stop_his_Rap1	CGCGGATCCCTAATGGTGATGGTGATGGTGGAGCAGCAGACATGATTTCT

Supplementary Table 4. Statistical analysis: Multiple comparison tests following ANOVA.

Figure	Result	Test	Comparison	Mean	95% confidence interval	Effect size	*t*	*q*	Degrees of freedom	Summary	Adjusted *P* value
**1a**	Binding (%)	Sidak's	PC PE PIP2 PS	53	[41;64]	10.7	9.892		16	****	9.6×10^{-8}
			PC PE	5	[0;10]						
			PC PE PIP2 PS Chol	66	[60;72]	1.3	2.781		16	*	0.0395
			PC PE PIP2 PS	53	[41;64]						
			PC PE PIP2 PS Chol + cAMP	73	[60;85]	1.1	1.368		16	ns	0.4688
			PC PE PIP2 PS Chol	66	[60;72]						
**1b**, left	Binding (%)	Dunnett's	PC PE PA	21	[21;22]	1.2		1.559	6	ns	0.2768
			PC PE	18	[11;25]						
			PC PE CL	17	[8;27]	1.0		0.1626	6	ns	0.9809
			PC PE	18	[11;25]						
**1b**, right	Binding (%)	Dunnett's	PC PE PA + cAMP	17	[3;30]	1.6		1.975	6	ns	0.1607
			PC PE + cAMP	27	[17;38]						
			PC PE CL + cAMP	40	[18;62]	1.5		2.423	6	ns	0.0887
			PC PE + cAMP	27	[17;38]						
**1c**	k_{cat}/K_M ($s^{-1}.M^{-1}$)	Dunnett's T3	cAMP	1.2×10^4	[0.8×10^4;1.6×10^4]	1.1	0.2928		19.51	ns	0.9874
			PM liposomes	1.3×10^4	[0.8×10^4;1.8×10^4]						
			cAMP + PM liposomes	8.9×10^4	[7.0×10^4;10.8×10^4]	7.4	8.764		10.93	****	8.0×10^{-6}
			cAMP	1.2×10^4	[0.8×10^4;1.6×10^4]						
			cAMP + PM liposomes	8.9×10^4	[7.0×10^4;10.8×10^4]	6.8	8.596		11.28	****	9.7×10^{-6}
			PM liposomes	1.3×10^4	[0.8×10^4;1.8×10^4]						

Supplementary Table 5. Statistical analysis: Two-tailed unpaired Student's *t* tests.

Figure	Result	Welch's correction	*t*	Degrees of freedom	Comparison	Mean	95% confidence interval	Effect size	Summary	*P* value
**1d**, left	k_{obs} (s^{-1})	No	0.1471	4	PC PE PA	1.3×10^{-3}	[2.2×10^{-5};2.5×10^{-3}]	1.0	ns	0.8902
					PC PE	1.2×10^{-3}	[1.5×10^{-4};2.3×10^{-3}]			
**1d**, right	k_{obs} (s^{-1})	No	2.456	4	PC PE PA + cAMP	3.6×10^{-3}	[3.1×10^{-3};4.1×10^{-3}]	1.3	ns	0.0700
					PC PE + cAMP	2.8×10^{-3}	[1.6×10^{-3};4.0×10^{-3}]			
**1e**, left	k_{obs} (s^{-1})	No	0.1512	4	PC PE CL	9.3×10^{-4}	[5.7×10^{-4};12.9×10^{-4}]	1.0	ns	0.8871
					PC PE	9.1×10^{-4}	[5.6×10^{-4};12.6×10^{-4}]			
**1e**, right	k_{obs} (s^{-1})	No	0.3178	4	PC PE CL + cAMP	2.5×10^{-3}	[2.3×10^{-3};2.7×10^{-3}]	1.0	ns	0.7665
					PC PE + cAMP	2.6×10^{-3}	[2.2×10^{-3};2.9×10^{-3}]			
**2b**	K_D (nM)	Yes	4.0082	28.001	Liposomes + cAMP	103	[30;176]	278	***	0.0004
					Solution + cAMP	28625	[14049;43201]			
**2c**	k_{max} (s^{-1})	Yes	18.91	31.87	Liposomes + cAMP	0.013	[0.012;0.014]	5.5	****	$< 10^{-15}$
					Solution + cAMP	0.0024	[0.0021;0.0027]			
**2e**	K_D (nM)	Yes	5.127	24.04	Liposomes + 007	15	[-4;33]	107	****	3.0×10^{-5}
					Solution + 007	1568	[943;2192]			
**2f**	k_{max} (s^{-1})	Yes	13.44	20.09	Liposomes + 007	0.032	[0.027;0.036]	12.3	****	1.7×10^{-11}
					Solution + 007	0.0026	[0.0023;0.0028]			
**4a**	Activity (%)	Yes	21.11	1.973	CE3F4	21	[-10;53]	4.7	**	0.0024
					DMSO	100	[65;135]			
**4d**, left	Activity (%)	No	0.7130	4	CE3F4	87	[41;134]	1.1	ns	0.5152
					DMSO	100	[39;161]			
**4d**, right	Activity (%)	No	13.64	4	CE3F4	50	[36;64]	2.0	***	0.0002
					DMSO	100	[92;108]			
**4e**, left	Activity (%)	Yes	0.09172	1.304	CE3F4	101	[11;191]	1.0	ns	0.9390
					DMSO	100	[65;136]			
**4e**, right	Activity (%)	Yes	9.577	1.808	CE3F4	62	[33;91]	1.6	*	0.0147
					DMSO	100	[59;141]			
**4f**, left	Binding (%)	No	1.455	4	CE3F4	81	[35;126]	1.2	ns	0.2193
					DMSO	100	[66;134]			
**4f**, right	Binding (%)	Yes	7.865	2.087	CE3F4	58	[35;81]	1.7	*	0.0140
					DMSO	100	[97;103]			
